# The effect of levamisole on kidney function in children with steroid-sensitive nephrotic syndrome

**DOI:** 10.1007/s00467-021-05231-4

**Published:** 2021-09-06

**Authors:** Lieke A. Hoogenboom, Hazel Webb, Kjell Tullus, Aoife Waters

**Affiliations:** 1 Department of Pediatric Nephrology, Great Ormond Street Hospital for Children NHS Foundation Trust, London, UK; 2 Nephro-Urology Unit, UCL GOS Institute of Child Health; Department of Pediatric Nephrology, Great Ormond Street Hospital for Children NHS Foundation Trust, London, UK

**Keywords:** Levamisole, Creatinine, eGFR, Nephrotoxicity

## Abstract

**Background:**

Levamisole is frequently used as a steroid-sparing agent in children with steroid-sensitive nephrotic syndrome. Side effects, such as neutropenia, gastro-intestinal upset and skin rash, have been reported. We noted an increase in creatinine in some of our patients, but literature on the effect of levamisole on kidney function is lacking.

**Methods:**

A retrospective cohort study was conducted, including patients 1–18 years of age, treated for steroid-sensitive nephrotic syndrome with levamisole at Great Ormond Street Hospital for Children between January 2010 and January 2020. Data was collected on clinical observations and serum creatinine values before, during and after treatment. eGFR was calculated using the Schwartz equation.

**Results:**

In total, 75 children were included in the analysis. The median duration of treatment was 19 (IQR 12–27) months. The median estimated GFR was 134 (IQR 119–160), 101 (IQR 91–113) and 116 (IQR 106–153) ml/min/1.73 m^2^, respectively, before, during and after treatment with levamisole. The difference between eGFR before and after treatment compared with during treatment was statically significant (*P* < 0.0001). During the treatment period, the eGFR decrease was not progressive. The median levamisole dose was 2.5 (IQR 2.3–2.6) mg/kg on alternate days, and the dose was not correlated with the decrease in eGFR (*r* = 0.07, 95% CI − 0.22 to 0.35).

**Conclusion:**

Levamisole significantly decreases eGFR. However, this decrease is not progressive or irreversible and would not be an indication to discontinue the treatment.

## Introduction

Levamisole is an immune-modulating imidazothiol-derived anthelminthic drug which is frequently used as a steroid-sparing agent in children with steroid-sensitive nephrotic syndrome. Previous research focused on its efficacy to reduce the number of relapses but also reported on its side effects. The most frequently described side effects are leucopenia/neutropenia (3.7%), gastro-intestinal upset (2.4%) and skin rash (1.5%). These symptoms are normally reversible upon discontinuing treatment [1]. Interestingly, to date, no study has been conducted reviewing the effect of levamisole on kidney function.

In our clinical population, we have, in a small number of children, seen a rise in serum creatinine after they were started on levamisole. The values normalised when we stopped the drug. There are no studies on the effect of levamisole on serum creatinine and estimated GFR (eGFR). Therefore, we conducted a retrospective cohort study reviewing the serum creatinine values in patients with steroid-sensitive nephrotic syndrome treated with levamisole at our hospital to review the magnitude and reversibility of this observation.

## Materials and methods

A retrospective cohort study was conducted, reviewing all patients 1–18 years of age, treated for steroid-sensitive nephrotic syndrome with levamisole at Great Ormond Street Hospital for Children (GOSH) between January 2010 and January 2020. Data were collected on creatinine values before, during and after treatment and haematology results in children treated with levamisole. When this data was unavailable at GOSH, data from the shared care hospital was retrieved. Patients were excluded if no creatinine value was available during the time of treatment.

The height of the children was used to calculate estimated GFR (eGFR) by using the Schwartz equation with the constant of 36.5. The dates of commencing and discontinuing levamisole were noted and so was the reason for stopping the drug; either no longer needed after 2 years of successful relapse control, due to side effect or because of insufficient relapse control and need to escalate the treatment to another steroid-sparing agent.

Statistical analysis was performed in GraphPad Prism V.9. Descriptive statistics were performed to analyse median and interquartile range, and the Wilcoxon matched-pairs signed rank test was used to analyse the difference in eGFR before, during and after levamisole treatment. *P*-values < 0.05 were considered significant.

## Results

A total of 127 children were treated with levamisole in our centre between January 2010 and January 2020. In 52 children, there were no laboratory data available during their time on levamisole treatment, and they were excluded. In total, 75 children were included for analysis. Laboratory data before starting and after discontinuing levamisole treatment was available in 55 and 39 children, respectively. In 29 cases, data were available on all three time points. Sixteen children were still on levamisole at the time of analysis. In 8 children, 5 before starting levamisole and 3 after discontinuing, no recent height was available to correlate with the creatinine, and therefore eGFR could not be calculated. The median age at start of levamisole treatment was 5 (IQR 4–7) years. There were more boys included (57 male vs. 18 female). The median duration of treatment was 19 (IQR 12–27) months with a median levamisole dose of 2.5 (IQR 2.3–2.6) mg/kg on alternate days. Creatinine values after treatment were obtained a median 4 (IQR 1–15) months after discontinuing levamisole. In 25 children, the treatment had been escalated to another steroid-sparing agent, such as tacrolimus, mycophenolate mofetil or cyclophosphamide, due to ongoing relapses. The levamisole dose was the same in children who responded to the treatment and those requiring escalation of treatment. None of the patients was taking an ACE inhibitor or on nephrotoxic medication, such as calcineurin inhibitor, while on levamisole.

The median serum creatinine before starting levamisole was 30 (IQR 24–36, *n* = 55) µmol/l. It increased to a median serum creatinine of 43.5 (IQR 35–50, *n* = 75) µmol/l (*P*-value < 0.0001) while on levamisole, and after discontinuing the drug, the serum creatinine was 40 (IQR 33–47.5, *n* = 39) µmol/l. Both the initial increase and the difference between creatinine while on treatment compared to after treatment was statistically significant with *P*-values of < 0.0001 and 0.0037, respectively.

**Fig. 1:**
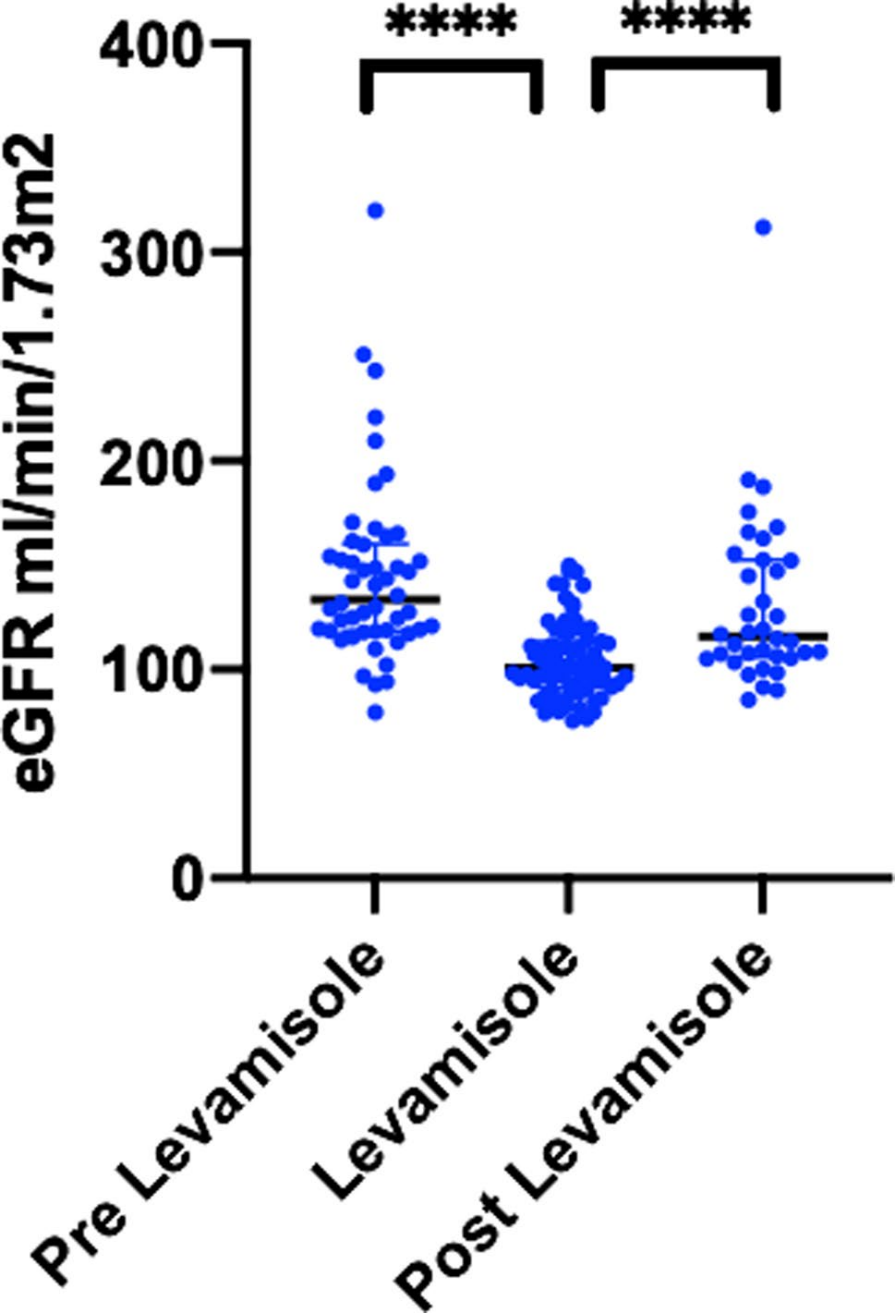
Estimated GFR (in ml/min/1.73 m^2^) before, during and after treatment with Levamisole. Black line indicates the median. **** *P*-value < 0.0001

The median eGFR before starting levamisole treatment was 134 (IQR 119–160, *n* = 50) ml/min/1.73 m^2^. On levamisole, this decreased to 101 (IQR 91–113, *n* = 75) ml/min/1.73 m^2^ and the eGFR increased after discontinuing levamisole to 116 (IQR 105–153, *n* = 36) ml/min/1.73 m^2^ (Fig. 1). The eGFR during levamisole treatment was significantly reduced compared to before and after treatment with *P*-values of < 0.0001. The difference between eGFR before and after levamisole did not reach statistical significance (*P* = 0.4047). The eGFR dropped shortly after starting treatment, it was not progressive, and there was no correlation between the reduction in eGFR and levamisole dose (*r* = 0.07, 95% CI − 0.22 to 0.35).

In 16 children, the eGFR during treatment dropped below 90 ml/min/1.73 m^2^. In these cases, the median eGFR before starting levamisole was 119 (IQR 116–141, *n* = 9), decreasing to 84 (IQR 80–86, *n* = 16) while on levamisole, and 106 (IQR 97–108, *n* = 7) ml/min/1.73 m^2^ after discontinuing the drug. One child had a reduced eGFR prior to starting treatment and normalised after levamisole was discontinued. The drop in eGFR in children with eGFR below 90 ml/min/1.73 m^2^ was 37 (IQR 28–54) as compared to 29 (IQR 14–55) in those whose eGFR remained above 90 ml/min/1.72m^2^.

In 8 patients, the levamisole was discontinued because of the development of side effects. Two children developed neutropenia (neutrophil count < 0.5 10^9/l) and three anaemia (Hb < 110 g/l), but only one of these children required discontinuing treatment. One child developed a rash and one an oral ulcer with neutropenia, both of whom improved after stopping the treatment. In 5 patients, the decrease in eGFR below 90 ml/min/1.73 m^2^ was the reason to stop levamisole treatment.

## Discussion

To our knowledge, this is the first study reporting on the effect of levamisole on kidney function in children with nephrotic syndrome while on treatment. Although there have been several studies reporting on side effects of levamisole, such as anaemia, neutropenia and skin rash, the literature on its effect on kidney function and creatinine clearance is scarce. There is one patient in the study by Gruppen et al. who developed an unexplained reduction in creatinine clearance which resolved after discontinuing levamisole [2]. Abeyagunawardena et al. reported no difference in serum creatinine between children on alternate day and daily dose of levamisole [3].

It can be assumed that the rise in observed creatinine is due to some impairment of kidney function; however, other possibilities need to be considered. There are at least three such possibilities that need to be explored: reduced hyperfiltration from successful treatment, an interference with the quantification method and inhibition of tubular excretion of creatinine and/or increased tubular reabsorption of creatinine.

Children who are in relapse of nephrotic syndrome generally show glomerular hyperfiltration [4], often seen as a creatinine value that is below the normal range for that child with a GFR that is higher than normal [5]. Branten et al. showed that, in hypoalbuminemia, the creatinine clearance is increased by the increased tubular excretion and/or reduced tubular reabsorption of creatinine [4]. The children in our cohort at the start of levamisole were in remission, although there was no data available on the time between remission and the serum creatinine value. Therefore, it can be argued that although the children were in remission when starting levamisole, the creatinine had not yet reached a steady state. Although this could contribute to the difference between eGFR before and during treatment with levamisole, it does not explain the normalising eGFR after discontinuing the drug.

To date, there is no literature reporting on the interference of levamisole with either the enzymatic creatinine quantification method or Jaffe method, nor do the manufacturers report this interference. Most of the creatinine values in our cohort were obtained by using the enzymatic method. When reviewing our cohort, there were 2 children for which the laboratory at the local hospital used the Jaffe method. These children, though small in number, show the same trend in creatinine rise as compared to the enzymatic method. Research will be required to exclude interference from either levamisole or its metabolites with more certainty.

Tubular excretion of creatinine in healthy individuals contributes to 10–40% of the total excretion [6, 7]. Several drugs, such as trimethoprim, cimetidine and antiretroviral drugs, have been found to increase serum creatinine by inhibiting the enzymes involved in the tubular excretion of creatinine, such as OCT2, OAT2 and MATE1 [6]. There is no direct evidence that levamisole inhibits any one of these transporters in the kidney. However, Martel et al. showed an inhibitory effect of levamisole on the organic cation transporter rOCT1 in the liver [8]. Plante et al. found that the tubular excretion of thiamine by the organic cation transport system was reduced by levamisole [9]. Moreover, they found that glomerular inulin clearance was not affected by levamisole, indicating that glomerular excretion is not affected by levamisole. The creatinine changes that we found in our cohort, which were 28% increase upon starting treatment and 20% decrease after discontinuing, could be consistent with inhibition of tubular excretion of creatinine.

Regarding the possibility of nephrotoxicity of levamisole, our data show that, after starting the drug, the decrease in eGFR is not progressive over the treatment period (data not shown) and there is no correlation found between the treatment duration or dose and the decrease in eGFR. The latter is in keeping with results by Abeyagunawardena et al. who found no difference in serum creatinine between alternate day and daily dosing of levamisole. In our cohort, eGFR improves after discontinuing levamisole, which is consistent with previous research by Sümegi et al., who reported no difference in eGFR before and after levamisole treatment [10]. However, there is no direct evidence, such as urine biomarkers or histology, to prove or rule out nephrotoxicity caused by levamisole. Based on the available data, we would argue that a drop in eGFR secondary to levamisole would not necessitate discontinuing treatment, especially since the alternative treatments of tacrolimus or cyclophosphamide are proven nephrotoxic.

In conclusion, we found that levamisole significantly increases serum creatinine. Although this increase is not progressive or irreversible, the exact mechanism is unknown. There is circumstantial evidence in the literature suggesting that levamisole inhibits the tubular excretion of creatinine, but further research would be required to gain insight into the underlying mechanisms involved.

## References

[1] Muhlig AK, Lee JY, Kemper MJ, Kronbichler A, Yang JW, Lee JM, Shin JI, Oh J (2019). Levamisole in children with idiopathic nephrotic syndrome: clinical efficacy and pathophysiological aspects. J Clin Med.

[2] Gruppen MP, Bouts AH, Jansen-van der Weide MC, Merkus MP, Zurowska A, Maternik M, Massella L, Emma F, Niaudet P, Cornelissen EAM, Schurmans T, Raes A, van de Walle J, van Dyck M, Gulati A, Bagga A, Davin JC, all members of the Levamisole Study Group (2018). A randomized clinical trial indicates that levamisole increases the time to relapse in children with steroid-sensitive idiopathic nephrotic syndrome. Kidney Int.

[3] Abeyagunawardena AS, Karunadasa U, Jayaweera H, Thalgahagoda S, Tennakoon S, Abeyagunawardena S (2017). Efficacy of higher-dose levamisole in maintaining remission in steroid-dependant nephrotic syndrome. Pediatr Nephrol.

[4] Branten AJ, Vervoort G, Wetzels JF (2005). Serum creatinine is a poor marker of GFR in nephrotic syndrome. Nephrol Dial Transplant.

[5] Carrie BJ, Golbetz HV, Michaels AS, Myers BD (1980). Creatinine: an inadequate filtration marker in glomerular diseases. Am J Med.

[6] Chu X, Bleasby K, Chan GH, Nunes I, Evers R (2016). Transporters affecting biochemical test results: creatinine-drug interactions. Clin Pharmacol Ther.

[7] Omote S, Matsuoka N, Arakawa H, Nakanishi T, Tamai I (2018). Effect of tyrosine kinase inhibitors on renal handling of creatinine by MATE1. Sci Rep.

[8] Martel F, Ribeiro L, Calhau C, Azevedo I (1999). Inhibition by levamisole of the organic cation transporter rOCT1 in cultured rat hepatocytes. Pharmacol Res.

[9] Plante GE, Erian R, Petitclerc C (1981). Renal excretion of levamisole. J Pharmacol Exp Ther.

[10] Sumegi V, Haszon I, Ivanyi B, Bereczki C, Papp F, Turi S (2004). Long-term effects of levamisole treatment in childhood nephrotic syndrome. Pediatr Nephrol.

